# Phylotranscriptomics supports numerous polyploidization events and phylogenetic relationships in *Nicotiana*

**DOI:** 10.3389/fpls.2023.1205683

**Published:** 2023-07-28

**Authors:** Shuaibin Wang, Junping Gao, Zhaowu Li, Kai Chen, Wenxuan Pu, Chen Feng

**Affiliations:** ^1^ Tobacco Research Institute of Technology Centre, China Tobacco Hunan Industrial Corporation, Changsha, China; ^2^ Puai Medical College, Shaoyang University, Shaoyang, China; ^3^ Jiangxi Provincial Key Laboratory of ex-situ Plant Conservation and Utilization, Lushan Botanical Garden, Chinese Academy of Sciences, Jiujiang, China

**Keywords:** *Nicotiana* L., transcriptomic analysis, polyploid formation, evolutionary relationship, divergence time

## Abstract

**Introduction:**

*Nicotiana* L. (Solanaceae) is of great scientific and economic importance, and polyploidization has been pivotal in shaping this genus. Despite many previous studies on the *Nicotiana* phylogenetic relationship and hybridization, evidence from whole genome data is still lacking.

**Methods:**

In this study, we obtained 995 low-copy genes and plastid transcript fragments from the transcriptome datasets of 26 *Nicotiana* species, including all sections. We reconstructed the phylogenetic relationship and phylogenetic network of diploid species.

**Results:**

The incongruence among gene trees showed that the formation of *N. sylvestris* involved incomplete lineage sorting. The nuclear–plastid discordance and nuclear introgression absence indicated that organelle capture from section *Trigonophyllae* was involved in forming section *Petunioides*. Furthermore, we analyzed the evolutionary origin of polyploid species and dated the time of hybridization events based on the analysis of PhyloNet, sequence similarity search, and phylogeny of subgenome approaches. Our results highly evidenced the hybrid origins of five polyploid sections, including sections *Nicotiana, Repandae, Rusticae, Polydicliae*, and *Suaveolentes*. Notably, we provide novel insights into the hybridization event of section *Polydicliae* and *Suaveolentes*. The section *Polydicliae* formed from a single hybridization event between maternal progenitor *N. attenuata* and paternal progenitor *N. undulata*; the *N. sylvestris* (paternal progenitor) and the *N. glauca* (maternal progenitor) were involved in the formation of section *Suaveolentes*.

**Discussion:**

This study represents the first exploration of *Nicotiana* polyploidization events and phylogenetic relationships using the high-throughput RNA-seq approach. It will provide guidance for further studies in molecular systematics, population genetics, and ecological adaption studies in *Nicotiana* and other related species.

## 1 Introduction

*Nicotiana* L. (Solanaceae) is of great scientific and economic importance, containing the cultivated tobaccos (*N. tabacum* and *N. rustica*), the model plant (*N. benthamiana*), as well as some essential ornamentals (e.g., *N. alata* and *N. sylvestris*) (Wang and Bennetzen, 2015). The genus *Nicotiana* comprises about 87 species, including one recently reported new Australian species in the section *Suaveolentes* (*N. paulineana*) (Bally et al., 2021), nearly half of which are allotetraploids (Knapp et al., 2004). The classification of *Nicotiana* mainly relied on geographical distribution, morphological characters, and cytological investigations, which were first reported by Goodspeed (1956). It was subsequently updated by Knapp et al. (2004) based on the phylogenetic analysis and morphological description. The current classification of *Nicotiana* comprises three subgenera (*Rustica*, *Tabacum*, and *Petunioides*) and 13 sections, five of which contain polyploids formed by interspecific hybridization (seven diploid sections: *Alatae*, *Noctiflorae*, *Petunioides*, *Undulatae*, *Paniculatae*, *Trigonophyllae*, *Tomentosae*, and five polyploidy sections: *Suaveolentes*, *Repandae*, *Nicotiana*, *Polydicliae*, *Rusticae*) (Knapp et al., 2004; Leitch et al., 2008). The *Nicotiana* species are distributed across tropical and temperate regions and are primarily endemic to South America, North America, and Australia (Knapp et al., 2004), of which *N. tabacum* is one of the most widely cultivated non-food crops, having been spread worldwide by humans. About 75% of *Nicotiana* species occur naturally in America and 25% in Australia (Aoki and Ito, 2000; Clarkson et al., 2004). Interestingly, all native Australian *Nicotiana* species belong to section *Suaveolentes* (Bally et al., 2021). In addition, *Nicotiana* species exhibit a spectacular range of floral morphology and color, genome size, and karyotypic diversity (Leitch et al., 2008; Marks et al., 2011; Renny-Byfield et al., 2013; McCarthy et al., 2015; McCarthy et al., 2016) and the polyploids of *Nicotiana* formed at different stages of evolutionary divergence (Leitch et al., 2008). The genus *Nicotiana* is, therefore, an excellent system in which to take advantage of recent advances in the research of speciation, biodiversity, and phytogeography (Aoki and Ito, 2000; McCarthy et al., 2016).

Phylogenetic relationships have been the subject of study in this genus for around two decades based on the plastid markers (coding and noncoding) (Aoki and Ito, 2000; Clarkson et al., 2004), low-copy nuclear genes (Kitamura et al., 2001; Clarkson et al., 2017), nuclear ribosomal internal transcribed spacer (ITS) (Chase et al., 2003) and random amplified polymorphic DNA (RAPD) analysis (Khan and Narayan, 2007). The previous phylogenetic studies have provided new insights into interspecific relationships (Clarkson et al., 2004) and also led to a modification in the traditional classification of the genus *Nicotiana* (Knapp et al., 2004). However, these phylogenetic analyses are based on only several molecular markers or short sequences (glutamine synthetase gene, leafy/floricaula gene, ITS, *trnL-F*, *trnS-G*, *ndhF*, and *matK*) and provide limited resolution of relationships among *Nicotiana* species. And the deep relationships of *Nicotiana* have usually neither been resolved nor well-supported. Recent analyses using the complete chloroplast genomes of *Nicotiana* in 11 sections have recovered a nearly fully resolved phylogenetic relationship and deduced a potential maternal progenitor of polyploid species (Wang et al., 2022). However, evidence of phylogenetic relationships and diploid progenitors of polyploid species from the nuclear are still lacking. Therefore, sufficient evidence from whole genome data is needed to deduce the deep phylogenetic relationships and demonstrate the phylogenetic discordance among this genus.

Polyploidization has been pivotal in shaping this genus (Leitch et al., 2008; McCarthy et al., 2016). Approximately half of the *Nicotiana* species were considered natural tetraploid species of different ages (Goodspeed, 1956; Knapp et al., 2004; Leitch et al., 2008). The majority of the *Nicotiana* species possess 12 or 24 chromosome pairs, except for several diploid species in section *Alatae* with 9 or 10 pairs and several polyploidy species in section *Suaveolentes* with 15, 16, 18, 19, 20, 21, or 22 pairs (Khan and Narayan, 2007; Marks et al., 2011). So far, the morphological, distributional, cytogenetic, and molecular evidence has been used to discover the diploid progenitors for each tetraploid species (Chase et al., 2003; Lim et al., 2005; Kelly et al., 2013; Schiavinato et al., 2020). One of the most studied polyploids in this genus was the tetraploid *N. tabacum* (2n = 4x = 48), with different lines of evidence suggesting *N. sylvestris* (2n = 2x = 24, maternal donor) and *N. tomentosiformis* (2n = 2x = 24, paternal donor) as candidate parents based on morphological observation (Goodspeed, 1956), plastid genome comparison (Yukawa et al., 2006), RAPD analysis (Khan and Narayan, 2007), the genome size (Leitch et al., 2008), and whole genome sequencing (Sierro et al., 2014; Sierro et al., 2018). In addition, the species in section *Repandae* were proposed to have a hybrid origin between *N. sylvestris* and *N. obtusifolia* based on a phylogenetic context (Leitch et al., 2008). The species in the section *Rusticae* were suggested as hybrids between the ancestral species of *N. paniculata* of section *Paniculatae* and *N. undulata* of section *Alatae* based on the comparison of karyotype and genome size (Khan and Narayan, 2007; Leitch et al., 2008). The ancestral species of section *Suaveolentes* possibly were related to sections *Acuminatae* (which should be called *Petunioides*, following Knapp et al., 2004), *Noctiflorae*, and *Alatae* based on external morphology (Khan and Narayan, 2007). Still, this hypothesis has never been formally tested (Leitch et al., 2008), and the ancestry of allopolyploid species in section *Polydicliae* is unresolved (Khan and Narayan, 2007; Leitch et al., 2008). Thus, a genome-wide perspective on the origin and evolution of allopolyploid species, including estimation of divergence dates, has been lacking.

The rise of high-throughput sequencing techniques has produced massive amounts of genomic or transcriptomic data, providing an unprecedented opportunity for systematic and evolutionary studies in great depth (Lemmon and Lemmon, 2013). Notably, because of the relatively low cost of transcriptome sequencing compared with genome sequencing and the fact that phylotranscriptomics is almost as reliable as phylogenomics (Cheon et al., 2020), phylotranscriptomic analysis has emerged as the preferred method for studying evolutionary biology (Cheon et al., 2021). Recent studies based on nuclear genes, especially phylotranscriptomics, have been successful in resolving relationships of various scales from the genus (Yang et al., 2018; Chen et al., 2021) to angiosperm-wide (Yang et al., 2020), even gymnosperms (Liu et al., 2022) and ferns (Qi et al., 2018) plants. During the last decade, large amounts of transcriptomic data have been generated for this genus, which has provided new opportunities for studying the phylogenetic relationships and evolution of polyploids at the scale of whole genomes. The newer approaches based on transcriptomic studies applying to *Nicotiana* species will provide a more accurate evaluation of speciation and polyploid events of *Nicotiana* (Clarkson et al., 2017).

*Nicotiana* species have significant economic importance. Most notably, tobacco (*N. tabacum*) is a major cash crop widely used in the production of tobacco products (Wang and Bennetzen, 2015). Additionally, *Nicotiana* species have pharmaceutical and research value. They are extensively used in the preparation of medicines, including both traditional herbal remedies and modern pharmaceuticals (Wang and Bennetzen, 2015). Moreover, due to the rich genomic and genetic diversity of *Nicotiana* species, they serve as ideal model organisms for studying genetic engineering, molecular biology, and genetics (Wang and Bennetzen, 2015).

In this study, we obtained the transcriptome datasets from 26 *Nicotiana* species, including 17 diploid species in eight sections and nine allopolyploid species in five sections. Two outgroup species (*Petunia axillaris* and *Petunia inflata*) from the genus *Petunia* were used as the sister taxa in the family of Solanaceae. We performed the analysis of phylogeny and PhyloNet based on the low-copy nuclear genes and transcript fragments of plastid genomes, respectively. Our study aimed to address the following topics: (1) to re-examine the classification and phylogenetic relationships reported in previous studies of *Nicotiana* and provide a relationship strongly supported among the lineages of *Nicotiana*; (2) to assess the conflict between nuclear and plastid phylogenetic topology and the inconsistency of gene trees; (3) to investigate the potential parental origin for the species of five polyploid sections; (4) to estimate the divergence time of diploid species and the time of the interspecific hybridization events that gave rise to polyploid species. This study represents the first exploration of *Nicotiana* phylogeny and timing of diversification in allopolyploids utilizing the high-throughput RNA-seq approach. It will provide valuable insights for future research in molecular systematic, population genetic, and ecological adaptation studies in *Nicotiana* and other related species.

## 2 Materials and methods

### 2.1 Data source, reads trimming, and transcriptome assembly

Here the transcriptome datasets were obtained from 26 species of genus *Nicotiana* including 17 diploid *Nicotiana* species and nine *Nicotiana* allotetraploid species representing each of the 13 *Nicotiana* sections: *Alatae* (n = 12), *Nicotiana* (n = 24), *Noctiflorae* (n = 12), *Paniculatae* (n = 12), *Petunioides* (*Noctiflorae*) (n = 12), *Polydicliae* (n = 24), *Repandae* (n = 24), *Rusticae* (n = 24), *Suaveolentes* (n = 16-24), *Sylvestres* (n = 12), *Tomentosae* (n = 12), *Trigonophyllae* (n = 12), and *Undulatae* (n = 12) (Clarkson et al., 2004). These transcriptome sequencing reads were retrieved from the NCBI Sequence Read Archive (SRA) database using the fastq-dump software of the SRA Toolkit package (available from https://github.com/ncbi/sra-tools). Additionally, two species of the genus *Petunia* (*P. axillaris* and *P. inflata*) from the non-*Nicotiana* Solanaceae family were included as outgroups. The protein-coding sequences of *P. axillaris* and *P. inflata* (Bombarely et al., 2016) were retrieved from Solanaceae Genomics Network (available from https://solgenomics.net/).

For transcriptome analyses, the quality control of raw data was performed using the fastp v0.23.2 (Chen et al., 2018), and the adapter, short reads (min. read length 50 bp), reads containing N, and reads with low-quality score (min. quality 20) were removed. All subsequent analyses were based on the filtered clean data. The clean data was first aligned into the genome sequences of allotetraploid *N. tabacum* (version 4.5, available from https://solgenomics.net/ftp/genomes/Nicotiana_tabacum/edwards_et_al_2017/) (Edwards et al., 2017) using STAR v2.7.10a (Dobin et al., 2012) with the default parameters. The aligned BAM files from the same species were merged, sorted, and indexed using samtools v1.15.1 (Danecek et al., 2021). Then, the quality of BAM files were assessed using samtools with the ‘flagstat’ and ‘depth’ algorithms. The Trinity v2.14.0 (Grabherr et al., 2011) was used to perform genome-guided *de novo* transcriptome assembly based on the BAM file of each species with default parameters. The BUSCO v5.3.2 (Manni et al., 2021) was applied to evaluate the completeness of the assembly results based on the solanales homologous gene database (available from https://busco-data.ezlab.org/v5/data/lineages/). The TransDecoder v5.5.0 (available from http://transdecoder.github.io) was employed to predict the open reading frame (ORF) within the assemblies with a minimum protein length of 100 bp. A BLAST search against the Uniref90 proteins database (Suzek et al., 2007) using BLAST (Camacho et al., 2009) and an HMMER search against the Pfam protein domain database v35.0 to identify common protein domains using PfamScan (Mistry et al., 2020). TransDecoder leveraged the outputs generated above to ensure that those peptides with blast hits or domain hits were retained in the set of reported likely coding regions. Then, CD-HIT v4.8.1 (Huang et al., 2010) was applied to remove redundant sequences with a threshold value of 0.95 identities. The statistic of assembly result was evaluated using ‘TrinityStats.pl’ script in the Trinity utility (Grabherr et al., 2011).

### 2.2 Identification and filtering of orthogroups

The OrthoFinder v2.5.4 (Emms and Kelly, 2015) was employed to infer orthologous genes from the predicted non-redundant CDS of the *Nicotiana* species and outgroups with default settings. Its utility has been tested in previous phylogenomic studies of plants (Peng et al., 2020) and animals (Fukushima and Pollock, 2020). To further increase the robustness of phylogenetic analyses, we filtered the orthologous groups according to the following criteria: (1) the max gene number of each sample in any orthologous group < 5; (2) the max gene number of each diploid sample in any orthologous group < 3; (3) the sequences of coding regions with the length >300 bp; (4) coverage of at least 50% of the 26 samples in each orthologous group. Thus, a total of 995 low-copy orthologous were generated to investigate *Nicotiana*’s phylogenetic relationships. The gene number and the presence/absence of orthologous groups in each species were shown as a heatmap using Python script.

A significant difficulty in reconstructing phylogeny was that polyploidization events involved many species across the genus *Nicotiana*, resulting in hidden paralogs (remaining single-copy genes after the loss of distinct paralogs in different taxa), which should be avoided in phylogenetic analysis. Thus, low-copy orthologous from only the diploid species were selected to build the phylogenetic backbone structure. The low-copy orthologous groups, including polyploid species, were used to construct the phylogenetic network and infer the hybrid process.

### 2.3 Diploid gene tree and species tree reconstruction

The polyploid samples were excluded from the filtered low-copy orthologous groups. Thus, 17 *Nicotiana* ploidy species and two *Petunia* species as outgroups with one-to-one single-copy orthologous were extracted for phylogenetic analyses. The concatenation- and coalescent-based methods were applied to reconstruct *Nicotiana*’s gene and species tree, respectively. For the concatenation-based method, individual single-copy orthologous were aligned using MAFFT v7.490 (Katoh and Standley, 2013) with the ‘L-INS- I’ algorithm, and poorly aligned regions were removed using Gblock v0.91b (Talavera and Castresana, 2007) with parameters ‘-b4 = 5 -b5=h -t=d’. All trimmed alignments were concatenated as a supermatrix and then performed summary statistics using AMAS (Borowiec, 2016). Two conventional approaches were used to construct a phylogenetic tree: (1) A maximum-likelihood (ML) analysis was performed using RAxML-NG v1.1.0 (Kozlov et al., 2019), with 1,000 bootstraps to find the best-scoring ML tree and the best substitution model GTR+I+G4 under the Akaike information criterion (AIC) calculated by ModelTest-NG v0.1.7 (Darriba et al., 2019). (2) A Bayesian-inference (BI) analysis was performed using MrBayes v3.2.7a (Ronquist et al., 2012), with the parameters: nst = 6 and rates = gamma. Four independent Markov chain Monte Carlo (MCMC) chains were run for 1,000,000 generations with random initial trees. Trees were sampled per 100 generations. The first 25% of trees were discarded as burn-in, with the remaining trees being used for generating the consensus tree. Tracer v1.7.1 (available from http://beast.community/tracer) was used to assess the quality of the MCMC simulations and the stability of runs. For the coalescent-based method, a massively parallel tool ParGenes v1.2.0 (Morel et al., 2018; Zhang et al., 2018; Kozlov et al., 2019) was used for model selection. And species tree inference based on the single-copy orthologous with the parameters applied as: ‘pargenes.py -a coalescence -o pargenes -c 32 -d nt -m –use-astral -b 1000’. Finally, the concatenated and coalescent phylogenetic results were visualized using FigTree v1.4.4 (available from http://tree.bio.ed.ac.uk/software/figtree/).

### 2.4 Identification of conflict and concordance among gene trees

Phyparts v0.0.1 (Smith et al., 2015) was employed to summarize the conflict and concordance information by comparing the gene tree of each single-copy orthologous against the species tree described above. Specifically, the gene tree of each single-copy orthologous with bootstrap support (BS) values was exported from the outputs of ParGenes and optimally rooted with the outgroups (*P. axillaris* and *P. inflata*) using phyx v1.3 (Brown et al., 2017). Still, in cases where the outgroups were missing, the gene trees were rooted by Minimal Ancestor Deviation (MAD) using MADroot (Tria et al., 2017). The branches in each gene tree with less than 33% BS were considered uninformative and collapsed using Newick utilities (Junier and Zdobnov, 2010). Two hundred four remaining gene trees and the species tree were used as inputs for phyparts to summarize the conflict and concordance information. The results of support and conflict statistics between gene trees and species trees were visualized with phypartspiecharts.py (available from https://github.com/mossmatters/phyloscripts/).

Furthermore, to quantify branch support values for the species tree, Quartet Sampling v1.3.1 (QS) (Pease et al., 2018) analysis was conducted with 1,000 replicates, and the log‐likelihood cutoff was 2. QS was a method to analyze molecular phylogeny by calculating branch support using repeated sampling of quartets. It provided four values in the outputs: QC (the Quartet Concordance score), QD (the Quartet Differential score), QI (the Quartet Informativeness score), and QF (the Quartet Fidelity score). The QS outputs were visualized as a figure with plot_QC_ggtree.R (available from https://github.com/ShuiyinLIU/QS_visualization). The results from phyparts and QS could provide alternative evidence for evaluating the discordance of gene trees.

### 2.5 Phylogenetic network analysis of diploid species

Nuclear-plastid discordance indicated a hybrid origin for section *Petunioides*, comprising *N. acuminata*, *N. miersii*, and *N. attenuata* (see Results). Moreover, the discordance of species and gene trees also suggested a hybridization or incomplete lineage sorting (ILS) for the section *Sylvestres* (see Results). We then used the maximum pseudolikelihood (MPL) approach implemented in PhyloNet v3.8.2 (Wen et al., 2018) to assess corroborative evidence supporting these conclusions. A total of 596 gene trees covering all 17 *Nicotiana* ploidy species and two outgroups were used to infer the phylogenetic network with the command ‘InferNetwork_MPL’ (Yu and Nakhleh, 2015). The section *Sylvestres* and section *Petunioides* were set as a potential hybrid, respectively. Uncertain nodes were bypassed in the gene trees by applying a bootstrap support threshold of 30 using the -b flag. The networks were visualized using Dendroscpoe v3.82 (Huson and Scornavacca, 2012).

In addition, the NeighborNet method implemented in SplitsTree v4.18.2 (Huson and Bryant, 2005) were used to reconstruct phylogenetic networks for the concatenated dataset. The K2P model was used for distance analysis, and support values at each node were estimated by running 1,000 bootstrap replicates.

### 2.6 Inference on the origin of allopolyploid species

Two strategies were used to infer the hybridization process within polyploid species of *Nicotiana*: (1) Inference based on the phylogenetic network. The phylogenetic network of each allopolyploid species was carried out using PhyloNet v.3.8.2 (Wen et al., 2018) with the command ‘InferNetwork_MP_Allopp’ under the MDC criterion (Yan et al., 2021). Network searches were performed using only nodes in the gene trees with BS support of at least 30%, allowing for one hybridization event. The networks were visualized using Dendroscpoe v3.82 (Huson and Scornavacca, 2012). (2) Inference based on the phylogenetic relationship of subgenomes of the allopolyploid. The first step was to split the CDS sequences of each allopolyploid species into two subsets based on the sequence similarity between polyploid and all diploid species. Specifically, each low-copy orthologous group was used to perform a sequence similarity search using BLAST+ (Camacho et al., 2009) against itself with the default parameters. The diploid species with the best hit of the CDS sequences in each allopolyploid species were recorded using the custom Python script from the blast tabular output. The total number of diploid species with the best hit in the CDS sequences of each polyploid species was counted and visualized as a heatmap. The diploid species most closely related to the progenitors of each allopolyploid would yield the highest number of best hits. Therefore, we sought to identify the diploid progenitors’ representatives for each allopolyploid. Then, each CDS sequence of allopolyploid species was assigned to the maternal or paternal group based on the putative results from the heatmap and previous PhyloNet analysis. The CDS sequences with ambiguous classification were discarded. The second step was to reconstruct the phylogenetic relationship based on the subgenomes of the polyploid species. Specifically, the phylogenetic relationship was inferred based on the concatenated datasets of the CDS sequences of diploid species and the classified CDS sequences of polyploid species using the ML analysis method described above. In addition, the inferred hybridization among the section *Sylvestres* and section *Petunioides* was also evaluated based on the sequence similarity search in the second strategy described above.

### 2.7 Plastid transcript assembly and phylogenetic analysis

Plastid genomes inherit maternally in the genus *Nicotiana* (Svab and Maliga, 2007). Thus the plastid phylogenetic tree could be used to determine the maternal progenitor of tetraploid species. Concretely, the clean reads were mapped into a previously published plastid genome (*N. sylvestris*: NC_007500.1) (Yukawa et al., 2006) with only one copy of inverted repeat regions using STAR v2.7.10a (Dobin et al., 2012), following the unmapped reads were filtered using samtools v1.15.1 (Danecek et al., 2021) with the parameter: -F 12. Genome-guided *de novo* transcriptome assembly was performed based on the filtered BAM file of each species. CD-HIT v4.8.1 (Huang et al., 2010) was used to exclude similar sequences with the parameter: -c 0.95. The local collinear regions among the filtered assemblies were identified using Mugsy v1.2.3 (Angiuoli and Salzberg, 2011). Only the conserved collinear regions with a length > 100 bp and coverage of at least 50% of the samples were extracted from the output of Mugsy. The filtered collinear regions were aligned and filtered according to the above-mentioned method. Then the combined supermatrix was used to perform phylogeny analysis using RAxML-NG v1.1.0 (Kozlov et al., 2019) with 1,000 bootstraps.

### 2.8 Dating of the divergence time and interspecific hybridization event

The divergence times within the species tree were inferred using BEAST v.2.6.7 (Bouckaert et al., 2019) optimized for OpenGL graphics. The dating analysis of concatenated single-copy genes of diploid species and the classified genes of allopolyploid species was performed with a strict clock, HKY substitution model, gamma site heterogeneity model, estimated base frequencies, and an ML starting tree. A Calibrated Yule model was specified as the tree prior. As no reliable fossils were available to calibrate the internal nodes of the *Nicotiana*, one secondary calibration from a recently published dated phylogeny of the Solanaceae (Särkinen et al., 2013) was used to calibrate the crown age of Solanaceae at 30.4 (95% HPD 26.3-34.0) million years ago (Ma). Two independent MCMC analyses of 10 million generations with 10% burn-in and sampling every 1,000 generations were conducted to evaluate the credibility of posterior distributions of parameters. The log files from BEAST were analyzed with Tracer v.1.7.0 (available from http://beast.community/tracer) to evaluate and ensure convergence, effective sample size (ESS) values, density plots, and trace plots. A maximum clade credibility tree with median heights was generated with TreeAnnotator v.1.8.4 (Bouckaert et al., 2019). The final tree was visualized using FigTree v1.4.4.

## 3 Results

### 3.1 RNAseq data, transcriptomes assembly, and ortholog identification

Here, a total of ~300 Gb transcriptome data from 26 *Nicotiana* species were collected, covering all 13 *Nicotiana* sections (**Table 1**). After read trimming, the number of clean reads per species ranged from 29.4 million to 454.6 million, with an average of 139.2 million.

**Table 1:** Summary information of the 26 *Nicotiana* species and the genome-guided transcriptome assemblies.

Species	Section	Ploidy level	Number of clean reads	Rate of properly mapping	Total number of trinity transcripts	Total length of contigs (bp)	Number of unigenes	GC content (%)	N50 of unigenes (bp)	BUSCO score
*N. acuminata*	*Petunioides*	diploid	279,341,354	75.59%	96,809	59,036,752	32,407	43.17	975	55.40%
*N. amplexicaulis*	*Suaveolentes*	tetraploid	44,726,966	76.82%	124,846	79,200,669	34,582	43.07	978	52.60%
*N. attenuata*	*Petunioides*	diploid	311,000,849	77.39%	134,204	90,057,835	28,812	42.45	1,515	96.60%
*N. benthamiana*	*Suaveolentes*	tetraploid	122,804,829	85.04%	234,682	188,327,938	71,557	42.49	1,242	95.80%
*N. bonariensis*	*Alatae*	diploid	55,090,758	88.53%	186,220	116,471,091	42,432	42.83	963	60.20%
*N. cavicola*	*Suaveolentes*	tetraploid	51,118,833	80.36%	131,916	87,576,621	35,640	43.01	1,059	60.30%
*N. clevelandii*	*Polydicliae*	tetraploid	51,559,778	86.65%	323,807	185,462,084	63,141	42.85	927	64.90%
*N. cordifolia*	*Paniculatae*	diploid	103,447,274	83.90%	201,692	134,731,202	41,923	42.62	1,098	70.80%
*N. glauca*	*Noctiflorae*	diploid	99,004,138	87.97%	243,441	183,438,304	54,161	42.63	1,047	70.50%
*N. knightiana*	*Paniculatae*	diploid	197,180,374	84.75%	220,167	198,049,253	48,534	42.6	1,263	84.00%
*N. miersii*	*Petunioides*	diploid	190,154,848	74.46%	77,747	45,701,251	29,068	43.4	897	51.50%
*N. noctiflora*	*Noctiflorae*	diploid	217,788,050	83.63%	208,075	122,281,149	41,406	42.62	1,056	67.80%
*N. obtusifolia*	*Trigonophyllae*	diploid	210,487,655	75.06%	85,016	50,875,955	28,194	43.3	963	50.00%
*N. otophora*	*Tomentosae*	diploid	36,545,666	66.86%	85,009	56,383,761	26,388	43.19	1,155	58.10%
*N. paniculata*	*Paniculatae*	diploid	454,631,909	83.46%	216,763	148,555,716	38,348	42.68	1,227	77.80%
*N. petunioides*	*Noctiflorae*	diploid	42,827,560	81.29%	108,580	66,927,564	33,327	43.06	981	54.80%
*N. plumbaginifolia*	*Alatae*	diploid	56,375,891	85.87%	168,392	106,350,036	45,195	42.74	933	61.80%
*N. raimondii*	*Paniculatae*	diploid	46,982,714	76.73%	111,544	85,905,570	31,998	42.92	1,170	66.50%
*N. rosulata*	*Suaveolentes*	tetraploid	84,859,415	82.19%	157,340	111,804,498	38,078	42.88	1,107	67.90%
*N. rustica*	*Rusticae*	tetraploid	84,391,418	83.68%	166,515	97,606,255	39,238	43.06	996	59.30%
*N. stocktonii*	*Repandae*	tetraploid	29,444,062	76.11%	112,297	73,805,927	35,211	43.12	984	53.60%
*N. sylvestris*	*Sylvestres*	diploid	302,837,182	91.04%	242,196	337,818,150	35,627	43.07	1,254	71.10%
*N. tabacum*	*Nicotiana*	tetraploid	248,141,718	93.84%	319,020	283,849,802	58,908	42.47	1,179	81.70%
*N. tomentosiformis*	*Tomentosae*	diploid	151,687,921	76.97%	170,353	136,584,817	35,233	42.78	1,293	77.20%
*N. undulata*	*Undulatae*	diploid	121,444,311	81.36%	158,486	97,525,737	38,222	42.93	1,047	64.70%
*N. velutina*	*Suaveolentes*	tetraploid	52,835,465	77.55%	138,048	103,844,488	34,822	42.96	1,158	67.10%

For the genome-guide transcript assembly, the clean reads of each species were mapped to the *N. tabacum* genome, which resulted in an average of 81.6% primary mapped reads, with the fewest in *N. otophora* (66.9%) and the most in *N. tabacum* (93.8%) (**Table 1**). These assemblies produced between 77,747 and 323,807 contigs (≥ 300 bp) for each species, with an N50 length ranging from 757 bp to 2,756 bp and a total length ranging from 45.7 Mb to 337.8 Mb (**Table 1**). A total of 26,388 to 71,557 unigenes were detected in each species, with N50 ranging from 897 to 1,515 bp and GC content from 42.4% to 43.4%. The assembly completeness evaluation results showed that all assembled unigenes had relatively high BUSCO scores, ranging from 50.0% (*N. obtusifolia*) to 96.6% (*N. attenuata*) among the 26 species (**Table 1**). These values, below a fully satisfactory BUSCO score, could be explained by the absence of tissue diversity and (or) enough data. Nevertheless, we considered that these genome-guided assembled gene sets would provide a reasonably good representation of the transcriptomes of *Nicotiana* species. For the plastid transcript assembly, the clean reads of each species were mapped to the plastid genome of *N. sylvestris*. The unmapped reads were filtered from the mapping results subsequently, which resulted in an average of 0.22 million mapped reads and an average of 85.6% reads coverage rate (>5x), with the fewest in *N. petunioides* (68.32%) and the most in *N. velutina* (99.7%) (**Supplementary Table S1**). After the transcript assemblies, ORF prediction, and de-redundancy, there were between 12 and 144 unigenes detected in each species, with N50 ranging from 873 to 19,600 bp and GC content ranging from 36.50% to 38.97% (**Supplementary Table S1**).

Based on the combined datasets from the unigenes of *Nicotiana* species and the outgroups, a total of 73,634 orthologous groups were identified, of which 995 low-copy genes passed our filtering criteria as mentioned above. Among these low-copy orthologous groups, the number of genes in each species ranges from 752 to 1,185. A total of 776 orthologous groups contained at least one of the outgroups, 558 shared in all 26 *Nicotiana* species, and 510 shared in all 28 species (26 *Nicotiana* specie and two outgroups) (**Supplementary Figure S1**). All the filtered low-copy genes were used to perform the phylogeny analysis, detect the parental progenitor, and date the hybridization event.

### 3.2 Diploid phylogenetic inference

We obtained the sequences of 995 genes with at least 273 bp in length for the nuclear phylogenetic dataset of diploid species. The alignment length for these single-copy genes ranged from 249 to 5,192 bp, with a mean length of 813 bp. After concatenation, the aligned 995-gene super matrix reached 808,952 bp in length, with 138,331 (17.1%) variable sites, 85,084 (10.5%) parsimony informative sites, and 154,142 (19.1%) missing sites (gaps and undetermined characters) (**Supplementary Table S2**).

**Figure 1:**
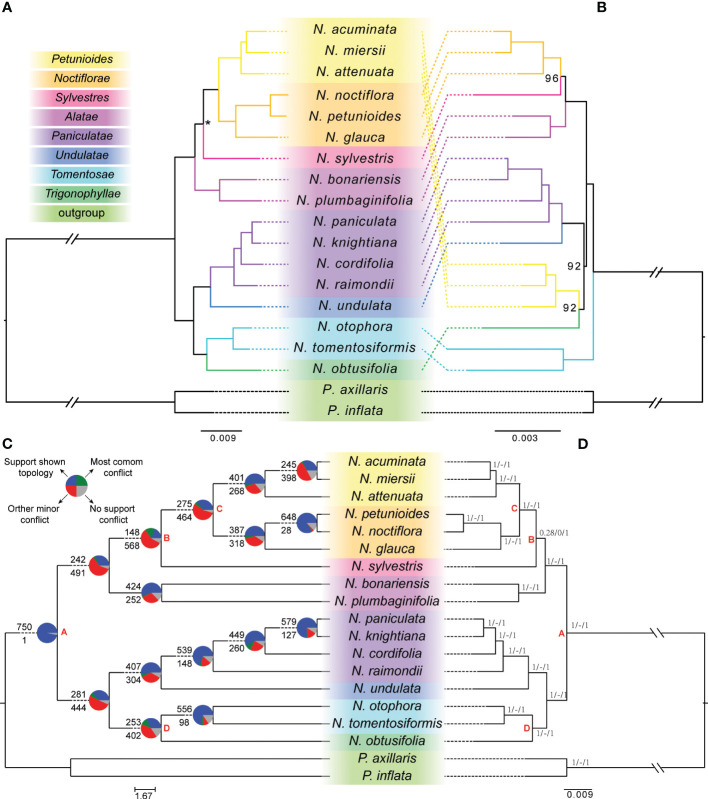
Tanglegram illustrating the nuclear-plastid discordance and the conflict signal among the gene trees in *Nicotiana* diploid species. **(A)** The diploid nuclear phylogeny recovered from concatenation- or coalescent-based methods based on the 995 single-copy genes (left). **(B)** The diploid plastid phylogeny recovered from the maximum-likelihood method based on the concatenated local collinear regions of transcript fragments (right). Bootstrap percentages were indicated beside the branches, and only values less than 100 were shown. **(C)** Patterns of gene-tree concordance and conflict of *Nicotiana* based on the phyparts analysis (left). The tree topology used was inferred by ASTRAL. The pie charts at each node show the proportion of genes in concordance (blue), conflict (green: a single dominant alternative; red: all other conflicting trees), and without enough information (gray). The numbers above and below each branch were the numbers of concordant and conflicting genes at each bipartition, respectively. **(D)** Information on *Nicotiana*’s gene-tree concordance and conflict based on the result from quartet sampling (right). Branch labels show quartet concordance (QC), quartet differential (QD), and quartet informativeness (QI), respectively, for each relationship. Corresponding clades in the nuclear and plastid phylogeny were colored. Asterisks on the branches of nuclear phylogeny (left) indicated local posterior probabilities of 0.94 in the coalescent-based species tree. Sections were classified according to Knapp et al. (2004) and labeled to the left.

For the nuclear phylogenetic dataset, the phylogenetic relationships reconstructed by ML and BI methods had identical topologies that separated the genus into two major clades with strongly supported (MLBS=100, PP=1.0) (**Figure 1A**). The first of them was integrated by a clade of section *Tomentosae*, sister of section *Trigonophyllae*, and a clade where section *Paniculatae* was sister to the clade of section *Undulatae*. The second was integrated by a clade, where section *Alatae* and *Sylvestres* were recovered as successive sister species of the clade of section *Petunioides* plus section *Noctiflorae* (**Figure 1A**). The coalescent-based species tree inferred from the diploid nuclear dataset yielded a concordant phylogenetic relationship (ASTRAL LPP=1.00) with the concatenation analysis, except for the *N. sylvestris* clade with a slightly less confident (ASTRAL LPP=0.94) (**Figure 1A**).

For the plastid phylogenetic dataset, the concatenated transcript fragment from plastid genomes reached 45,728 bp in length, with 2,103 (4.6%) variable sites, 1,125 (2.5%) parsimony informative sites, and 4,448 (9.72%) missing sites (**Supplementary Table S2**). The diploid plastid genomic phylogeny showed that the genus *Nicotiana* was monophyletic. Unlike nuclear phylogeny, within this plastid phylogenetic topology, three main clades with strong support were recovered (**Figure 1B**). The first was section *Tomentosae* as the basal-most clade. A clade of section *Petunioides* integrated the second, sister of section *Trigonophyllae*, and a clade of section *Paniculatae*, sister of section *Undulatae*. The third was integrated by a clade, where section *Alatae* and *Sylvestres* were recovered as successive sister species of section *Noctiflorae*.

Phylogenetic discordance was observed between topologies inferred from the nuclear and plastid concatenated datasets (**Figures 1A, B**). In nuclear phylogeny, section *Petunioides* (*N. acuminata*, *N. miersii*, and *N. attenuata*) were strongly supported (MLBS = 100) to be sister to section *Noctiflorae* (*N. noctiflora*, *N. petunioides*, and *N. glauca*). Section *Petunioides* were placed as sister to section *Trigonophyllae* (*N. obtusifolia*) with strongly supported (MLBS = 92) in plastid phylogeny. This result showed the hybridization of section *Petunioides* between section *Noctiflorae* and *Trigonophyllae*, which might explain the discordance of nuclear and plastid phylogenetic topologies. In addition, the section *Tomentosae* (*N. otophora* and *N. tomentosiformis*) was recovered as a basal taxon in plastid phylogeny. In construction, two main groups were recovered within the nuclear phylogeny.

### 3.3 Gene tree concordance and conflict

Our species tree inferred from coalescent nuclear data suggested that two major clades were identified in the phylogeny of the genus *Nicotiana* with full support (node A). Likewise, the result of Quartet Sampling (QS) demonstrated that node A was confirmed with full support (1/-/1; i.e., all informative quartets support that lineage) and phyparts result with almost all the informative gene trees (750 out of 751) support this topology (**Figures 1C, D**). Although the nuclear phylogenetic tree confirmed *N. sylvestris* sister to species of the clade of section *Petunioides* plus section *Noctiflorae* with full support, this clade (node B) was supported by only 28% of informative quartets with a skewed frequency for alternative discordant topologies (QS score = 0.28/0/1). Likewise, the result of phyparts supported this clade with only 148 out of the 716 informative gene trees (20.7%). This result revealed that ILS or hybridization might explain this phylogenomic discordance.

All the conflict nodes between nuclear and plastid phylogenetic topology showed relatively low informative gene trees supported in the phyparts result (**Figure 1C**). For example, nodes C and D have alternative discordant topologies. The result of phyparts supported this clade with only 275 of the 739 informative gene trees (37.2%) and 253 of the 655 informative gene trees (38.6%), respectively. In contrast, the QS result demonstrated that all these two nodes related to three sections were confirmed with full support (1/-/1) (**Figure 1D**).

### 3.4 Diploid phylogenetic networks

**Figure 2:**
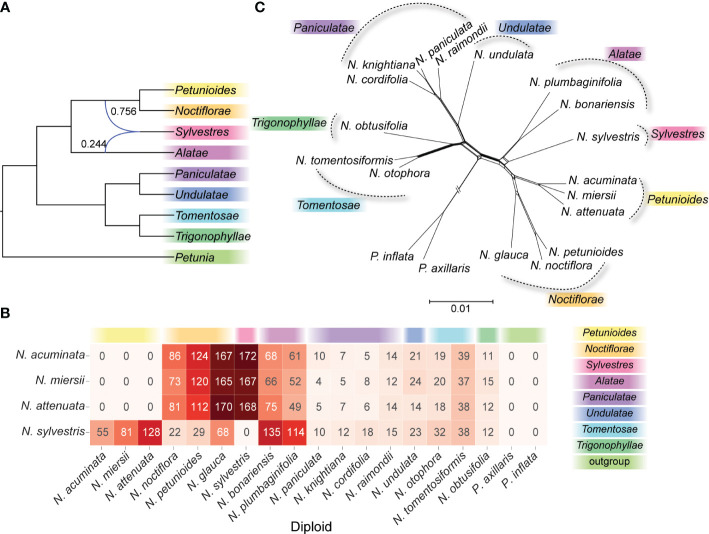
The phylogenetic network analysis of diploid species. **(A)** Phylogenetic networks were inferred by setting *N. sylvestris* as the hybrid species using the InferNetwork_ML method in PhyloNet. Blue branches indicated lineages involved in reticulated histories, and numerical values were the inheritance probabilities for each reticulation. **(B)** The heatmap showed the best hit number of the CDS sequences in *N. sylvestris* and section *Petunioides* against the other diploid species. **(C)** Phylogenetic networks generated by NeighborNet method in SplitTree4.

The phylogenetic discordances of nuclear-plastid and gene trees showed a complex evolutionary history among *N. sylvestris* and the species of section *Petunioides*. Using the *N. sylvestris* as potential hybrid species, our PhyloNet analysis indicated that *N. sylvestris* was a potential hybrid species between the section *Alatae* and the common ancestor of section *Noctiflorae* and *Petunioides*, or at least introgressed with section *Alatae* (**Figure 2A**). When using the species of section *Petunioides* as potential hybrid species, our PhyloNet analysis did not support the hybrid origin of *N. sylvestris* and the species of section *Petunioides* (data not shown). The analysis of sequence similarity search in *N. sylvestris* showed that a total of 264 genes in section *Petunioides*, 119 in section *Noctiflorae*, and 249 in section *Alatae* were similar to the gene sequences in *N. sylvestris* with the best hit (**Figure 2B**). In the sequence similarity search of section *Petunioides*, the best hit mainly occurred in *N. sylvestris*, section *Noctiflorae*, and *Alatae*, not in the section *Trigonophyllae* (**Figure 2B**).

Phylogenetic networks reconstructed using NeighborNet revealed apparent clustering among the *Nicotiana* diploid sections. When rooted in the genus *Petunia*, the topology of phylogenetic networks (**Figure 2C**) was very similar to the ML and Bayes trees (**Figure 1A**). However, *N. sylvestris* was alternative splits connecting to the section *Alatae* or the common ancestor of section *Petunioides* and *Noctiflorae* (**Figure 2C**), which showed uncertainty regarding the phylogenetic placement of this clade.

### 3.5 Putative diploid progenitors of polyploid species

**Figure 3:**
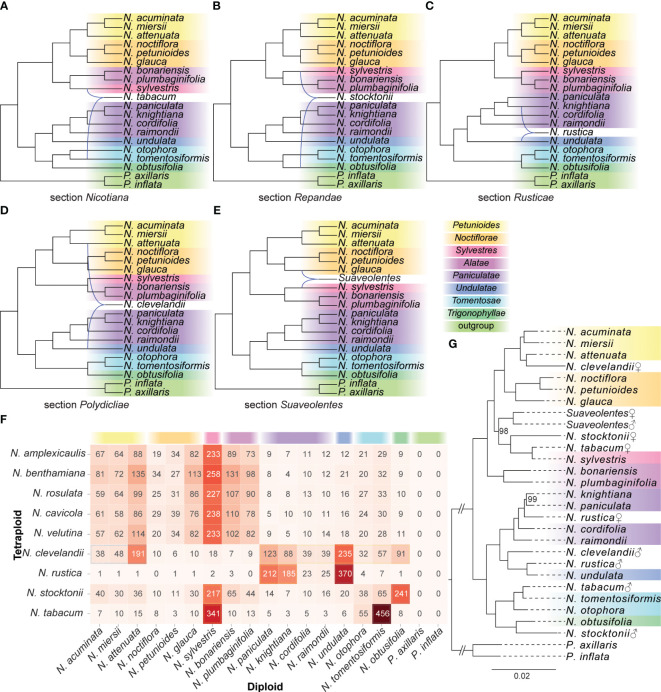
The diploid origin analysis of polyploid species. **(A–E)** Phylogenetic networks were inferred by setting each polyploid species as the hybrid species using the InferNetwork_MP_Allopp method in PhyloNet. Blue branches indicated lineages involved in reticulated histories. **(F)** The heatmap showed the best hit number of the CDS sequences in the polyploid species against the other diploid species. **(G)** The nuclear phylogeny recovered from the maximum-likelihood method based on the classified low-copy genes. Bootstrap percentages were indicated beside the branches, and only values less than 100 were shown. Sections were classified according to Knapp et al. (2004) and labeled to the right. ♀: maternal origin; ♂: paternal origin.

Based on the InferNetwork_MP_Allopp approach implemented in PhyloNet, we inferred the allopolyploid network from gene trees under the MDC criterion (**Figures 3A–E**). For the *N. tabacum* hybridization test, the PhyloNet result showed a hybridization event between the *N. sylvestris* and *N. tomentosiformis* (**Figure 3A**). For the *N. stocktonii*, the PhyloNet result showed a hybridization event between the *N. obtusifolia* and *N. sylvestris* (**Figure 3B**). For the *N. rustica*, the PhyloNet result showed a hybridization event between the *N. knightiana* and *N. undulata* (**Figure 3C**). For the *N. clevelandii*, the PhyloNet detected a hybridization between the *N. attenuata* and *N. undulata* into the clade *N. clevelandii* (**Figure 3D**). For the hybridization tests of the *N. velutina*, *N. cavicola*, *N. rosulata*, *N. benthamiana*, and *N. amplexicaulis*, the PhyloNet detected a hybridization between the *N. glauca* and *N. sylvestris* into the clade of section *Suaveolentes* (**Figure 3E**).

For each polyploid species, we counted the number of diploid species with the best hit of the CDS sequences (**Figure 3F**). In the CDS sequences of tetraploid *N. tabacum*, 341 and 456 CDS sequences had the best hit against the *N. sylvestris* and *N. tomentosiformis*, respectively, which suggested that *N. sylvestris* and *N. tomentosiformis* were the putative diploid progenitors of *N. tabacum*. Similarly, in tetraploid *N. Stocktonii*, a total of 217 and 241 CDS sequences against the *N. sylvestris* and *N. obtusifolia* with the best hit, respectively, suggested that *N. sylvestris* and *N. obtusifolia* were the putative diploid progenitors of *N. Stocktonii*. In the tetraploid *N. rustica*, 370 CDS sequences against the *N. sylvestris* with the best hit and 212 and 185 CDS sequences against the *N. paniculata* and *N. knightiana*, respectively, supported the hybrid simulation of *N. sylvestris* and *N. paniculata* and (or) *N. knightiana* into *N. rustica*. In the tetraploid *N. clevelandii*, 191 CDS sequences against the *N. attenuata* with the best hit. And 235 and 123 CDS sequences against the *N. undulata* and *N. paniculata*, respectively. This result supported the hybrid simulation between *N. attenuata* and *N. undulata* or *N. paniculata* into *N. clevelandii*. In the tetraploid *N. Velutina*, *N. cavicola*, *N. rosulata*, *N. benthamiana*, and *N. amplexicaulis*, 233–258 CDS sequences against the *N. sylvestris* with the best hit, and another parent could be origin from section *Petunioides*, *Noctiflorae* or *Alatae*.

Based on the classified sequences of polyploid species and transcript fragments of plastid, we construct the phylogenetic relationship of nuclear and plastid, respectively, including all diploid and polyploid species. In the plastid phylogenetic relationship (**Supplementary Figure S2**), the *N. tabacum* was sister to the *N. sylvestris*; the *N. rustica* was sister to the *N. knightiana*; the *N. clevelandii* was sister to the *N. obtusifolia*; both *N. stocktonii* and section *Suaveolentes* was sister to the section *Noctiflorae*. In the nuclear phylogenetic relationship (**Figure 3G**), the *N. tabacum* was sister to the *N. tomentosiformis* and *N. sylvestris*, respectively; the *N. clevelandii* was sister to the *N. undulata* and *N. attenuata*, respectively; the *N. stocktonii* was sister to the *N. obtusifolia* and *N. sylvestris*, respectively; and the *N. rustica* was sister to *N. undulata* and the common ancestor of *N. knightiana* and *N. paniculata*, respectively. The results of phylogenetic relationships provided clues about the parent origin of polyploid species. We concluded the hybridization event by combining the phylogenetic relationships of nuclear and plastid (**Figures 3G**, **S2**). Namely, the *N. tabacum* formed from a single hybridization event between extant relatives of maternal progenitor *N. sylvestris* and paternal progenitor *N. tomentosiformis*; the *N. stocktonii* formed from a single hybridization event between maternal progenitor *N. sylvestris* and paternal progenitor *N. obtusifolia*; the *N. rustica* formed from a single hybridization event between paternal progenitor *N. undulata* and maternal progenitor common ancestor of *N. knightiana* and *N. paniculata*; the *N. clevelandii* formed from a single hybridization event between maternal progenitor *N. attenuata* and paternal progenitor *N. undulata*; the section *Suaveolentes* could be formed from a single hybridization event between maternal progenitor *N. sylvestris* and paternal progenitor of *N. glauca*.

### 3.6 Dating the time of hybridization event among *Nicotiana* species

**Figure 4:**
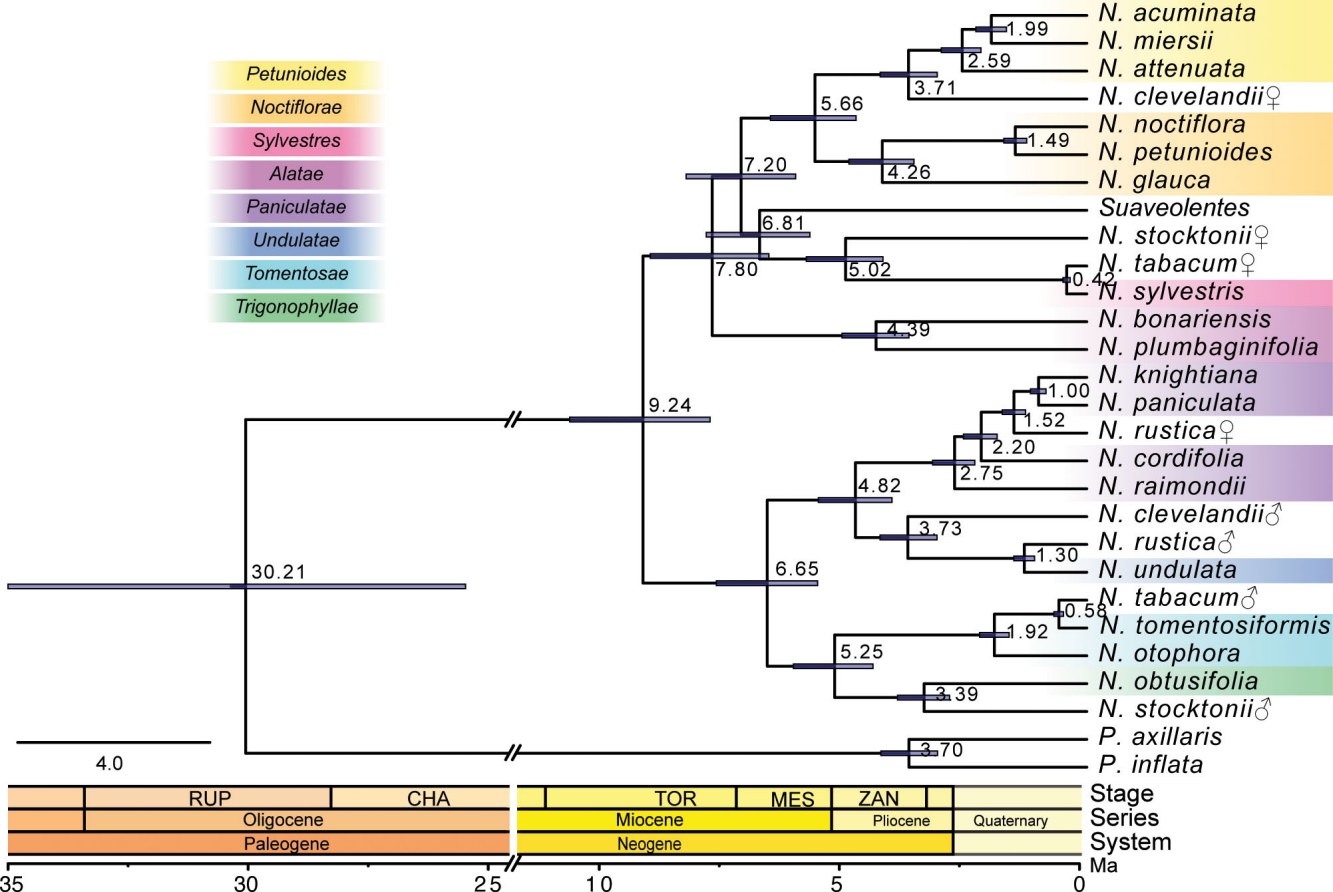
The phylogenetic tree showed the topology and divergence time for 26 *Nicotiana* species. Divergence times were indicated by light blue bars at the internodes; the range of these bars indicates 95% of the highest posterior density (HPD) interval of the divergence time. Numbers at the internodes indicate the mean divergence time. The geological timescale was illustrated at the bottom. ♀: maternal origin; ♂: paternal origin.

To date the time of hybridization events that had led to the formation of polyploid species, we then used the classified CDS based on the sequence similarity search to obtain the time distance between the hybrid and the parental species (a hybridization date) (**Figure 4**; **Supplementary Table S3**). The divergence between the genus *Nicotiana* and *Petunia* was dated to c. 30.2 Ma (95% Highest Posterior Density (HPD) = 25.6–35.2 Ma). The diversification of *Nicotiana* was inferred to begin at c. 9.24 Ma (95% HPD = 7.84–10.77 Ma). The *N. tabacum*, as the only species in section *Nicotiana*, was the youngest allotetraploid section with age estimates of c. 0.42 Ma from its maternal progenitor *N. sylvestris* (95% HPD = 0.35-0.50 Ma) and c. 0.58 Ma from its paternal progenitor *N. tomentosiformis* (95% HPD = 0.49–0.68 Ma). Section *Rusticae* was a monotypic section containing only *N. rustica* and yielded age estimates of c.1.52 Ma from its maternal progenitor, the common ancestor of *N. paniculata* and *N. knightiana* (95% HPD = 1.28-1.76 Ma) and c. 1.30 Ma from its paternal progenitor *N. undulata* (95% HPD = 1.09-1.52 Ma). Section *Polydicliae* consists of two species, of which *N. clevelandii* yielded age estimates of c. 3.71 Ma from its maternal progenitor, the common ancestor of section *Petunioides* (95% HPD = 3.11-4.30 Ma) and c. 3.73 Ma from its paternal progenitor *N. undulata* (95% HPD = 3.12-4.30 Ma). Section *Repandae* contained four species, of which *N. Stocktonii* yielded age estimates of c. 5.02 Ma from its maternal progenitor *N. sylvestris* (95% HPD = 4.25-5.84 Ma) and c. 3.39 Ma from its paternal progenitor *N. obtusifolia* (95% HPD = 2.86-3.94 Ma). Section *Suaveolentes* was the oldest and most species-rich allotetraploid section with age estimates of c. 6.81 Ma from its maternal progenitor, the common ancestor of *N. sylvestris* (95% HPD = 5.76-7.92 Ma).

## 4 Discussion

### 4.1 Strongly supported diploid phylogeny and nuclear-plastid phylogenetic discordance

The Molecular Phylogeny of the *Nicotiana* genus has been researched for more than two decades (Aoki and Ito, 2000). The previous phylogenetic analyses using a combination of the internal transcribed spacer (ITS) and several plastid markers suggested that section *Tomentosae* was the base taxa and the section *Petunioides* was sister to the MCRA of section *Noctiflorae*, *Alatae*, and Sylvestres (Leitch et al., 2008). Recently, the nuclear phylogenetic tree based on GS and LFY genes was used to investigate the timing of diversification (Clarkson et al., 2017). However, these phylogeny relationships in the genus *Nicotiana* were inferred from several nuclear or plastid makers, which lack enough support and reliable results. Our recent analysis of whole plastid genomes provided a well-supported phylogenetic relationship of 11 sections in *Nicotiana* (Wang et al., 2022), which supported the section *Tomentosae* as the base clade of all others, and the section *Petunioides* was sister to the section *Trigonophyllae*.

Over the past ten years, high‐throughput transcriptome sequencing has provided an unprecedented volume of available genetic data. The transcriptome data have been widely used for reconstructing the phylogenetic relationship of plants (Leebens-Mack et al., 2019) regardless of the tissue origin of the transcriptomes (Cheon et al., 2020). We used gene and species tree approaches to construct a diploid phylogeny of *Nicotiana* based on RNAseq data that includes representatives from all 13 sections recognized in the *Nicotiana* genus. Our phylogenetic analyses of nuclear and plastid datasets produced mostly harmonious and well‐supported relationships (i.e., ≥92 BP) among major lineages in *Nicotiana*, including those not well resolved in previous studies. According to the nuclear phylogeny, the earliest divergence in *Nicotiana* involves two major clades (**Figure 1A**). The first major clade was formed by the most recent common ancestor (MRCA) of sections *Trigonophyllae*, *Tomentosae*, *Undulatae*, and *Paniculatae*. Section *Trigonophyllae* was sister to section *Tomentosae*, while section *Undulatae* was sister to section *Paniculatae*. The second major clade was formed by the MRCA of sections *Petunioides*, *Noctiflorae*, *Sylvestres*, and *Alatae*. All of these relationships are strongly supported, except for the placement of section *Sylvestres* (ASTRAL LPP = 0.94/ASTRAL BS = 90/concatenated BS > 99; 148/568 informative gene trees). According to the plastid phylogeny, section *Tomentosae* was sister to all remaining sections, with a grade formed by the MRCA of section *Trigonophyllae*, *Petunioides*, *Undulatae*, *Paniculatae* and the MRCA of section *Alatae*, *Sylvestres*, and *Noctiflorae*.

Our nuclear analyses revealed two major clades, which were not indicated by previous molecular or morphological analyses (McCarthy et al., 2016; Clarkson et al., 2017). The plastid phylogenetic tree in this study showed a consistent placement with our previous result based on the whole plastid genomes (Wang et al., 2022). Furthermore, the availability of genome‐scale data allowed us to examine the consistency of phylogenetic signals in the nuclear and plastid genomes for the first time, and several incongruent have been identified between nuclear and plastid phylogeny, especially for section *Petunioides* and *Sylvestres* (**Figure 1**). Incomplete lineage sorting and hybridization are the two main evolutionary processes that could lead to incongruent topologies between nuclear and organelle genomes (Willyard et al., 2009; Kao et al., 2022). It is often difficult to disentangle these processes. For section *Sylvestres*, incomplete lineage sorting should count for the incongruent topologies between gene trees. In addition, our analyses show an inconsistent placement of section *Petunioides* between nuclear and plastid phylogeny (**Figures 1A, B**), which is likely the result of an interspecific hybrid origin of this section between section *Noctiflorae* and *Trigonophyllae* in more early time. In contrast, the hybrid signal and nuclear introgression were lacking in section *Petunioides* based on the sequence similarity search and PhyloNet analysis, respectively (**Figure 2B**), which means that organelle capture can explain this observation. Using multiple gene trees enabled us to detect evidence for hybridization events between diploid species and resolved the phylogeny more robustly than in the previous studies.

### 4.2 Inferring polyploid parentage of *Nicotiana* species

**Figure 5:**
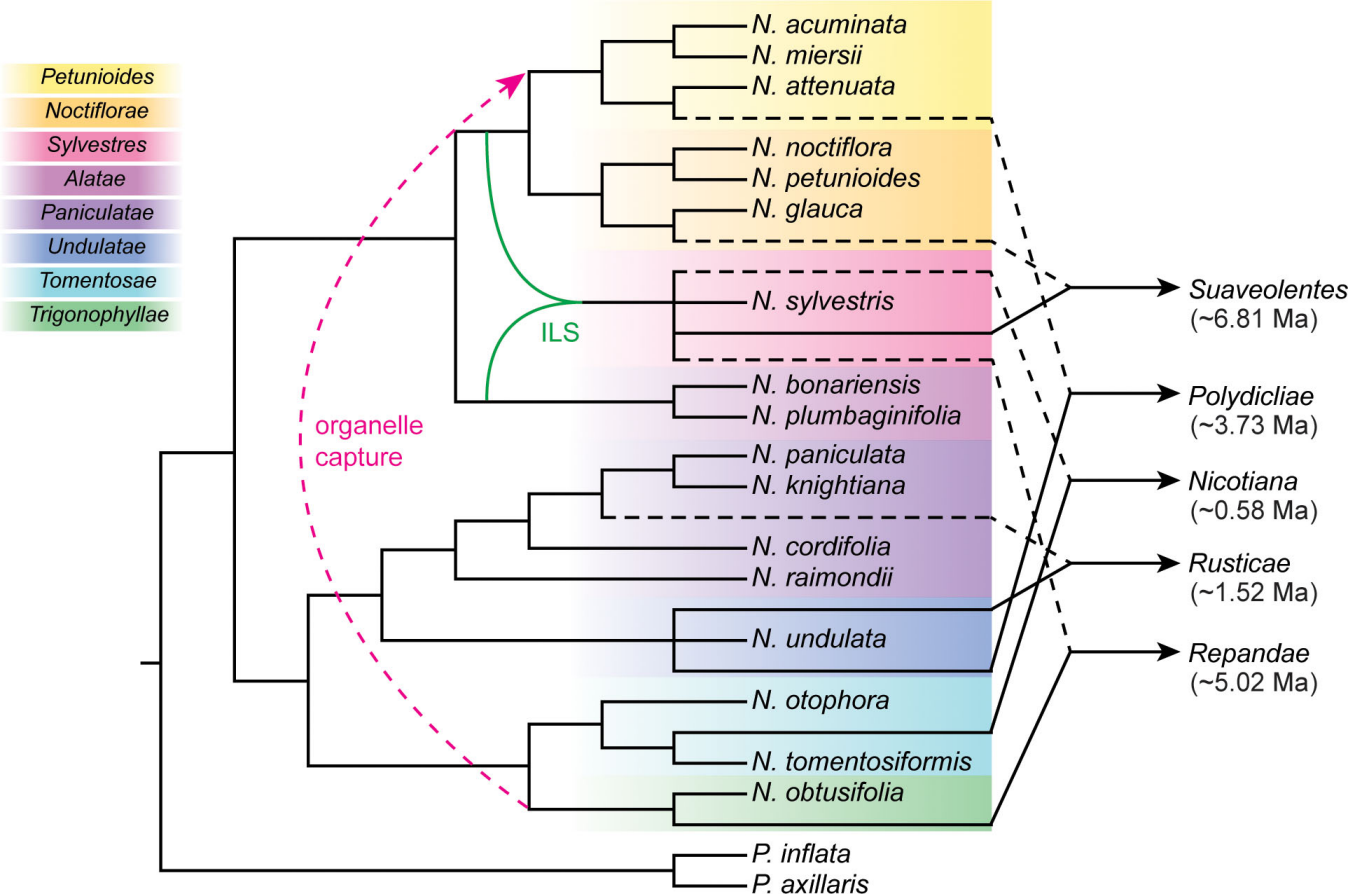
Cladogram summary of the polyploidization events and phylogenetic relationships in *Nicotiana*. Sections of allotetraploid origin were indicated by dashed black lines and solid black lines from their maternal lineages and paternal lineages, respectively. The section *Petunioides* involved organelle capture event was indicated by red dashed lines. The *N. sylvestris* involved incomplete lineage sorting (ILS) event was indicated by solid green lines. The time of hybridization events was noted under the allotetraploid sections. Sections were classified according to Knapp et al. (2004) and labeled to the left.

As the genus *Nicotiana* contains several groups of tetraploids that formed at different times from different diploid progenitors (Kelly et al., 2013), it provides an ideal system for understanding polyploidization, a pervasive and powerful evolutionary force in plants. We identified the most likely diploid progenitors of five allopolyploid sections using the combined approaches of the phylogenetic network, sequence similarity search, and phylogenetic tree of subgenomes (**Figure 3**). Our results provide novel insights into parental species for the *Nicotiana* polyploids. Below we discussed the putative parental species of *Nicotiana* polyploids and proposed the results obtained in this study (**Figure 5**).

#### 4.2.1 Section Nicotiana

*N. tabacum* (common tobacco), the only species in section *Nicotiana*, was most widely grown commercially for tobacco production. Its diploid ancestors (*N. sylvestris* as maternal progenitor and *N. tomentosiformis* as paternal progenitor) and the details of the hybridization have been well characterized (Sierro et al., 2013; Sierro et al., 2014). This study also provided three pieces of evidence to support that the diploid *N. sylvestris* and diploid *N. tomentosiformis* were the maternal and paternal progenitors of *N. tabacum*, respectively (**Figure 5**), which validated the reliability and accuracy of these strategies (**Figures 3A, G**).

#### 4.2.2 Section Repandae

Section *Repandae* consists of four allopolyploid species (*N. Nudicaulis*, *N. repanda*, *N. Stocktonii*, and *N. Nesophila*; 2n = 4x = 48) (Knapp et al., 2004; Clarkson et al., 2005). It has been thought that section *Repandae* formed from a single hybridization event between extant relatives of maternal progenitor *N. sylvestris* and paternal progenitor *N. obtusifolia*. Subsequently, four allopolyploid species were produced following speciation (Chase et al., 2003; Clarkson et al., 2010; Kelly et al., 2013; Dodsworth et al., 2017). Based on our strategies, the diploid *N. sylvestris* and *N. obtusifolia* were recognized as the maternal and paternal progenitors of section *Repandae*, respectively (**Figure 5**), consistent with the previous results (Dodsworth et al., 2017).

#### 4.2.3 Section rustica

Like common tobacco, *N. rustica* (Aztec tobacco) was the only species in section *Rusticae* and was an allotetraploid native to South America formed through a recent hybridization event (Knapp et al., 2004). Based on morphology, karyotype analyses, and breeding experiments, Goodspeed (1956) proposed that the diploid parents of *N. rustica* were ancestors of *N. paniculata* and *N. undulata.* Based on the comparative nuclear genome analysis, Sierro et al. (2018) found that the tetraploid species *N. rustica* inherited about 41% of its genome from its paternal progenitor, *N. undulata*, and 59% from its maternal progenitor, the common ancestor of *N. paniculata* and *N. knightiana*. Whereas, the pieces of evidence from comparative plastid genes and genome analysis revealed that the maternal parent of the tetraploid *N. rustica* was from the species of section *Paniculatae*, and the diploid *N. knightiana* was genetically closer than *N. paniculata* to *N. rustica* (Clarkson et al., 2004; Mehmood et al., 2020; Wang et al., 2022). The result from sequence similarity analysis support that both *N. knightiana* and *N. paniculata* might have donated the maternal genome of *N. rustica* (**Figure 3B**). Although the plastid genome of *N. knightiana* appears to be closer than that of *N. paniculata* to the *N.rustica* chloroplast genome, our analysis of PhyloNet and the phylogeny of subgenomes still suggested that a common ancestor of both *N. knightiana* and *N. paniculata* served as the maternal donor to *N. rustica* (**Figure 5**).

#### 4.2.4 Section Polydicliae

Section *Polydicliae* consists of two species, *N. quadrivalvis* and *N. clevelandii*, the only allopolyploid section found in western North America (Goodspeed, 1956). In early research, plastid-based analyses indicated that a diploid species from section *Trigonophyllae* was the maternal genome donor of section *Polydicliae* (Clarkson et al., 2004). Based on the analysis of genome size, Leitch et al. (2008) found that the genome size of *N. attenuata* was most closely related to the paternal genome donor of section *Polydicliae*. Clarkson et al. (2010) suggested that section *Polydiclieae* was the product of a cross between the ancestors of section *Trigonophyllae* (maternal) and *N. attenuata* (paternal). Based on the strict consensus trees from the ADH and LFY/FLO datasets, Kelly et al. (2013) proposed that section *Polydicliae* formed from a single hybridization event between extant relatives of maternal progenitor *N. obtusifolia* and paternal progenitor *N. attenuata*. Subsequently, the analysis of floral evolution in section *Polydicliae* was performed based on this hybridization model (McCarthy et al., 2015; Bombarely et al., 2016). Based on the phylogenetic tree of five plastid loci and two nuclear genes, Clarkson et al. (2017) suggested a similar result. However, we obtained a different conclusion. In the plastid phylogenetic relationship (**Supplementary Figure S2**), the *N. clevelandii* was sister to the *N. obtusifolia*, the plastid of which was transferred to section *Petunioides*. Thus, we now believe that section *Polydiclieae* is the product of a cross between the ancestors of *N. attenuata* (maternal) and *N. undulata* (paternal) based on our strategies (**Figure 5**).

#### 4.2.5 Section Suaveolentes

Section *Suaveolentes* was an almost all-Australian clade (the exception being *N. africana* of Namibia) of allopolyploid species, including the vital plant model *N. benthamiana* (Schiavinato et al., 2020). Karyotypic variation within this section was very enrichment from n = 15 to n = 32 chromosomes (Marks et al., 2011). It seems likely that section *Suaveolentes* has explosive radiation of taxa occurred, primarily accompanied by diploid reductions probably due to fusions of chromosomes (Clarkson et al., 2004). As the oldest *Nicotiana* polyploids (Clarkson et al., 2017), the diploid progenitors of section *Suaveolentes* were poorly understood (Marks et al., 2011). Goodspeed (1956) proposed that several diploid sections of *Nicotiana*, namely sections *Alatae*, *Sylvestres*, *Noctiflorae*, and *Petunioides*, were involved in the formation of the allotetraploid section *Suaveolentes*. Kelly et al. (2013) reconstructed the evolutionary origin of sect *Suaveolentes* using four regions from the nuclear and plastid genome. They identified a likely scenario where a member of the *N. Sylvestres* acted as the paternal progenitor, and a member of either section *Petunioides* or *Noctiflorae* was the maternal progenitor. Recently, Schiavinato et al. (2020) showed that the maternal progenitor of *N. benthamiana* was a member of section *Noctiflorae* and confirmed a member of section *Sylvestres* as a paternal subgenome donor. Our analysis based on the PhyloNet approaches supported a scenario where the *N. sylvestris* acted as the paternal progenitor, and the *N. glauca* of section *Noctiflorae* acted as the maternal progenitor in the formation of section *Suaveolentes* (**Figure 3E**), in line with previous findings (Schiavinato et al., 2020). But the analysis from the sequence similarity search and the phylogeny of the subgenome only support that *N. sylvestris* could have been involved in its formation (**Figure 3G**). Lastly, we summarized that the *N. sylvestris* (paternal progenitor) and the *N. glauca* (maternal progenitor) were involved in the formation of section *Suaveolentes* (**Figure 5**).

## 5 Conclusion

In conclusion, this study sheds light on the genetic diversity, phylogenetic relationships, and evolutionary history of *Nicotiana* species. The findings provide valuable insights into the classification and phylogenetic relationships within the genus. The identification of parental origins and the estimation of divergence times of polyploid species contribute to our understanding of speciation and hybridization events. Furthermore, the application of high-throughput RNA-seq technology in this study demonstrates its efficacy in phylogenetic studies and paves the way for future molecular systematic and population genetic investigations. The comprehensive dataset and analytical approaches used in this study serve as a valuable resource for further research in *Nicotiana* and related species.

## Data availability statement

The original contributions presented in the study are included in the article/**Supplementary Material**. Further inquiries can be directed to the corresponding author.

## Author contributions

SW and JG conceived and designed the study. SW and ZL conducted the bioinformatics analysis. ZL, WP, and KC assisted in data collection. SW, JG, and CF wrote and revised the manuscript. All authors read and approved the final manuscript.
