# Supplementary material for: Phylotranscriptomics supports numerous polyploidization events and phylogenetic relationships in Nicotiana
Source: Front Plant Sci. 2023 Jul 28;14:1205683. doi: 10.3389/fpls.2023.1205683 (PMC10421670; doi:10.3389/fpls.2023.1205683)
Supplement: Supplementary file 1 [file DataSheet_1.docx]

Supplementary Material

Phylotranscriptomics supports numerous polyploidization events and phylogenetic relationships in *Nicotiana*

Shuaibin Wang^1†^, Junping Gao^1†^, Zhaowu Li^3^, Kai Chen^1^, Wenxuan Pu^1^, Chen Feng^2*^

^1^Tobacco Research Institute of Technology Centre, China Tobacco Hunan Industrial Corporation, Changsha, China

^2^Jiangxi Provincial Key Laboratory of ex situ Plant Conservation and Utilization, Lushan Botanical Garden, Chinese Academy of Sciences, Jiujiang 332900, China

^3^Puai Medical College, Shaoyang University, Shaoyang 422099, China

^†^ These authors contributed equally to this work

^*^ **Correspondence:** Chen Feng: fengc@lsbg.cn

# Supplementary Figures and Tables

## Supplementary Figures


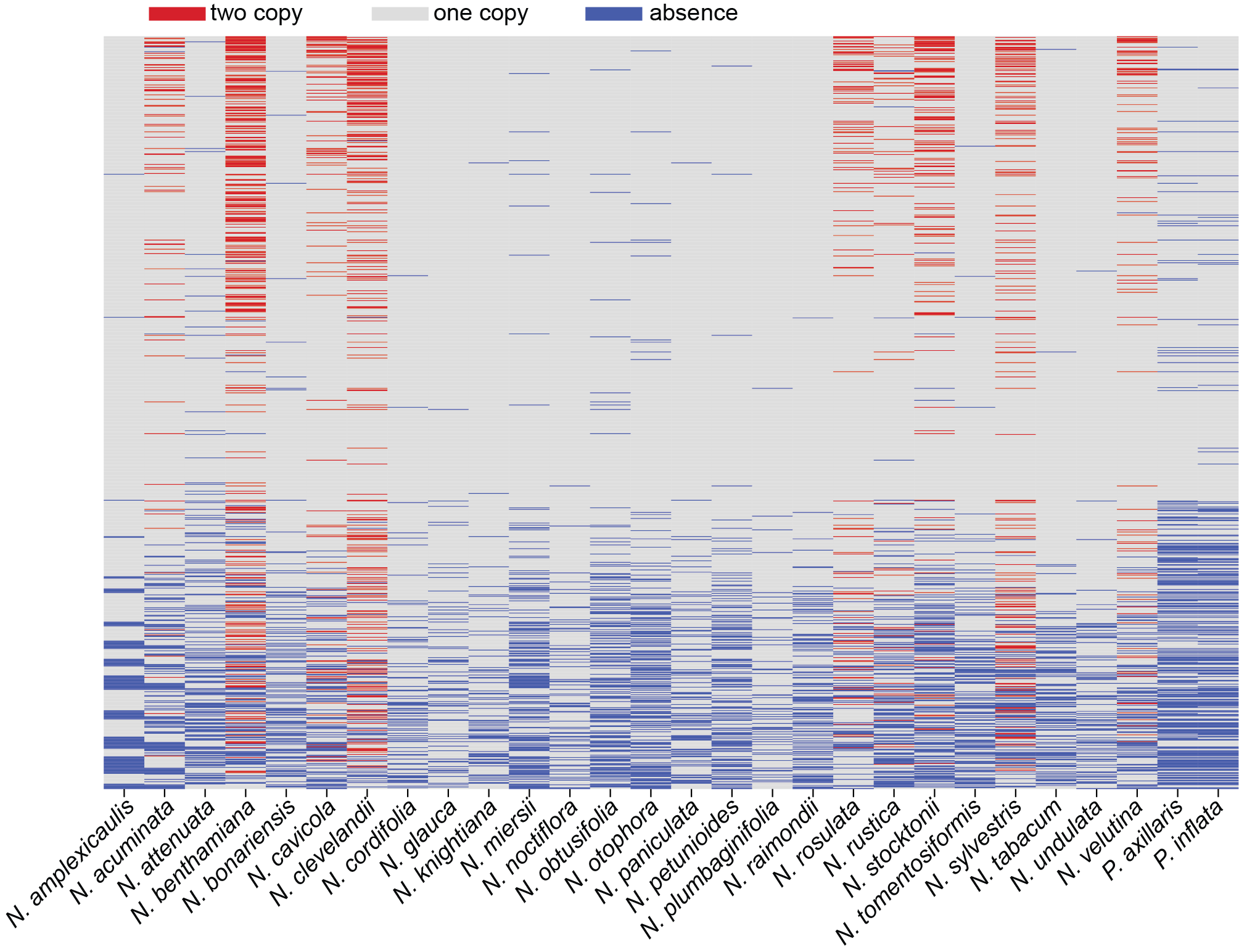


**Supplementary Figure 1.** Heatmap plot showing the gene copy number of each low-copy orthologous in the Nicotiana species. Red block: two copy genes in this orthologous; gray block: only one copy gene in this orthologous; blue block: no gene in this orthologous.


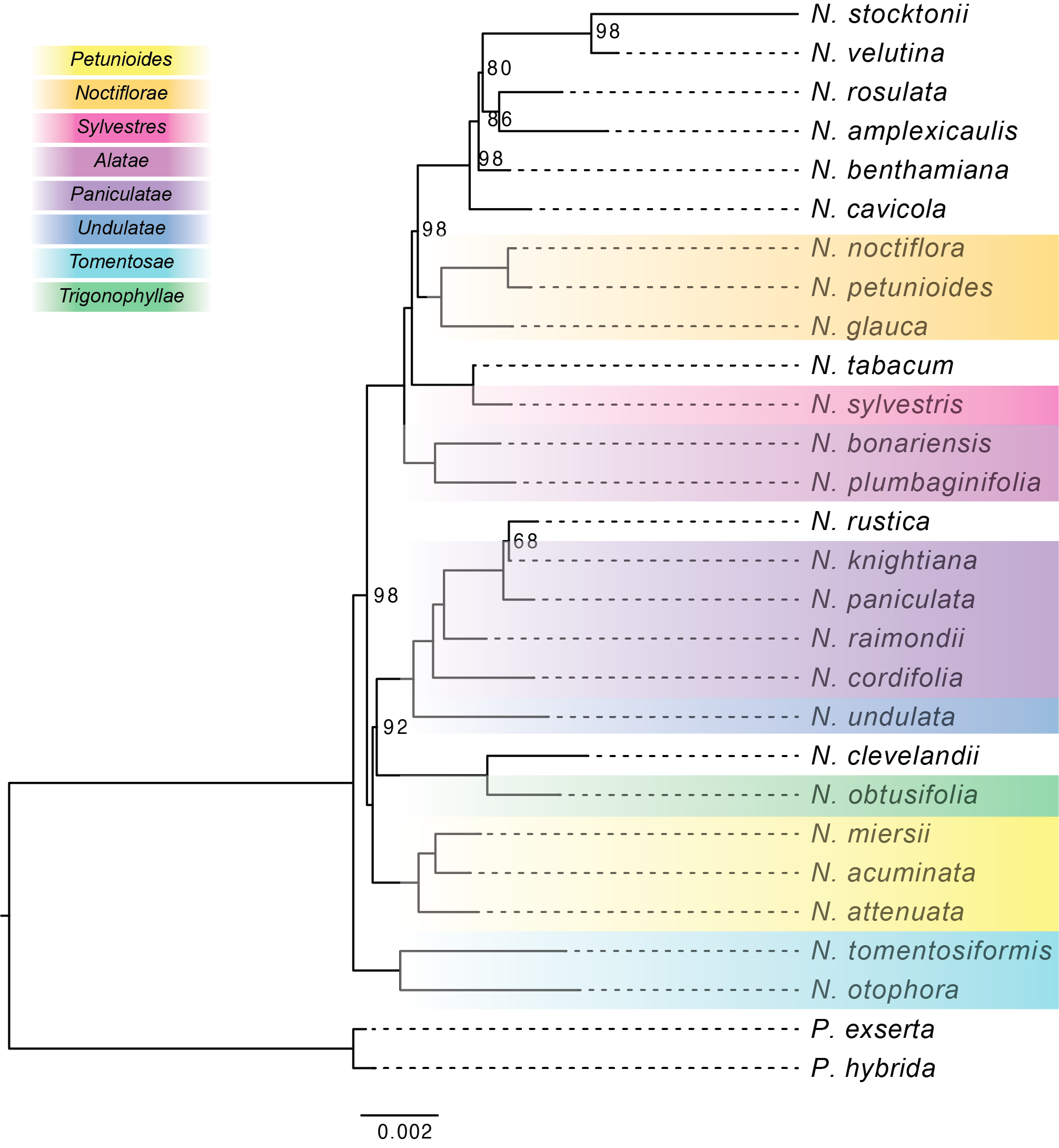


**Supplementary Figure 2.** The nuclear phylogeny recovered from the maximum-likelihood method based on the transcript fragments of plastid. Bootstrap percentages were indicated beside the branches, and only values less than 100 were shown. Sections were classified according to Knapp et al. (2004) and labeled to the left.

## Supplementary Tables

**Supplementary Table 1.** Summary of the data source and plastid transcriptome assembly.

| **Species** | **Data Source** | **Total length of contigs (bp)** | **Covreage rate (>5x )** | **Number of unigenes** | **unigenes N50 (bp)** | **GC content (%)** |
| --- | --- | --- | --- | --- | --- | --- |
| *N. acuminata* | SRR2913009/SRR2913011/SRR2913012 | 32,654 | 79.92% | 76 | 2,430 | 37.49 |
| *N. amplexicaulis* | SRR6516155 | 112,646 | 79.32% | 79 | 4,894 | 37.37 |
| *N. attenuata* | SRR2913026/SRR2913027/SRR2913028/SRR3596354 | 50,104 | 74.77% | 72 | 3,891 | 36.62 |
| *N. benthamiana* | SRR7540371/SRR7540372 | 108,792 | 97.52% | 19 | 12,681 | 37.06 |
| *N. bonariensis* | SRR6918813/SRR6918814/SRR6918815 | 43,596 | 93.98% | 90 | 2,762 | 37.35 |
| *N. cavicola* | SRR6516147 | 88,994 | 87.46% | 56 | 4,963 | 37.22 |
| *N. clevelandii* | SRR6918828/SRR6918829/SRR6918830 | 174,642 | 90.02% | 79 | 3,383 | 37.18 |
| *N. cordifolia* | SRR2106516 | 49,486 | 79.91% | 53 | 4,916 | 36.5 |
| *N. glauca* | SRR6918774/SRR6918777/SRR6918780/SRR6918783 | 282,393 | 71.91% | 135 | 873 | 37.73 |
| *N. knightiana* | SRR8169561/SRR8169572/SRR8169596/SRR8169569 | 57,300 | 85.24% | 16 | 13,730 | 37.32 |
| *N. miersii* | SRR2913071/SRR2913072 | 14,780 | 95.04% | 80 | 1,662 | 38.97 |
| *N. noctiflora* | SRR2106514 | 49,486 | 92.11% | 23 | 9,603 | 36.57 |
| *N. obtusifolia* | SRR2912992/SRR2912995/SRR2912996 | 874,904 | 91.63% | 64 | 4,422 | 36.78 |
| *N. otophora* | SRR6516159 | 381,632 | 71.58% | 51 | 7,698 | 36.69 |
| *N. paniculata* | SRR8169612 | 103,398 | 75.03% | 61 | 4,581 | 37.04 |
| *N. petunioides* | SRR6516150 | 74,766 | 68.33% | 99 | 2,736 | 37.31 |
| *N. plumbaginifolia* | SRR6918800/SRR6918799/SRR6918798 | 389,632 | 96.13% | 48 | 5,359 | 37.07 |
| *N. raimondii* | SRR6516154 | 135,714 | 76.77% | 51 | 5,704 | 36.83 |
| *N. rosulata* | SRR6516152 | 49,532 | 77.08% | 61 | 6,462 | 36.61 |
| *N. rustica* | SRR8169786 | 126,362 | 90.28% | 110 | 1,762 | 37.46 |
| *N. stocktonii* | SRR6516145 | 158,842 | 96.32% | 42 | 7,263 | 37.96 |
| *N. sylvestris* | ERR274390/ERR274391 | 238,291 | 71.11% | 144 | 1,335 | 38.12 |
| *N. tabacum* | SRR955771 | 372,332 | 93.05% | 20 | 8,697 | 37.08 |
| *N. tomentosiformis* | SRR2106531 | 1,233,726 | 90.99% | 12 | 18,721 | 37.51 |
| *N. undulata* | SRR8169535/SRR6516156 | 165,568 | 99.00% | 80 | 4,613 | 36.92 |
| *N. velutina* | SRR6516148 | 284,920 | 99.77% | 15 | 19,600 | 37.91 |

**Supplementary Table 2.** The alignment information of the supermatrix of nuclear genes and plastid transcript fragment.

| **Alignment name** | **Number of taxa** | **Alignment length (bp)** | **Missing percent** | **variable sites (%)** | **parsimony informative**  **(%)** | **GC content (%)** | **A** | **C** | **G** | **T** | **-** |
| --- | --- | --- | --- | --- | --- | --- | --- | --- | --- | --- | --- |
| 995-gene supermatrix of nuclear genes | 19 | 808,952 | 0.191 | 0.171 | 0.105 | 0.441 | 3,942,611 | 2,756,708 | 3,379,773 | 3,826,645 | 1,464,350 |
| Supermatrix of plastid transcript fragment | 19 | 45,728 | 0.097 | 0.046 | 0.025 | 0.372 | 239,766 | 151,531 | 140,059 | 252,964 | 84,512 |

**Supplementary Table 3.** Node ages of polyploid hybrid origins yielded by BEAST2 using data of subgenomes.

| **Hybrid** | **Section** | **Maternal progenitor** | **Maternal mean age (95% HPD interval)** | **Paternal progenitor** | **Paternal mean age (95% HPD interval)** |
| --- | --- | --- | --- | --- | --- |
| *N. tabacum* | *Nicotiana* | *N. sylvestris* | 0.42 (0.345-0.496) | *N. tomentosiformis* | 0.58 (0.486-0.682) |
| *N. stocktonii* | *Repandae* | *N. sylvestris* | 5.02 (4.245-5.839) | *N. obtusifolia* | 3.39 (2.857-3.938) |
| *N. rustica* | *Rusticae* | *N. paniculata* and *N. knightiana* | 1.52 (1.276-1.759) | *N. undulata* | 1.30 (1.090-1.517) |
| *N. clevelandii* | *Polydicliae* | *N. attenuata* | 3.71 (3.114-4.299) | *N. undulata* | 3.73 (3.120-4.304) |
| *Suaveolentes** | *Suaveolentes* | *N. sylvestris* | 6.81 (5.765-7.922) | - | - |

^*^The subgenome of species in section *Suaveolentes* was difficult to detect and the ages of origin of these hybrides has not been determined.
